# Contractile Properties of MHC I and II Fibers From Highly Trained Arm and Leg Muscles of Cross-Country Skiers

**DOI:** 10.3389/fphys.2021.682943

**Published:** 2021-06-16

**Authors:** Kasper Degn Gejl, Lars G. Hvid, Erik P. Andersson, Rasmus Jensen, Hans-Christer Holmberg, Niels Ørtenblad

**Affiliations:** ^1^Department of Sports Science and Clinical Biomechanics, University of Southern Denmark, Odense, Denmark; ^2^Department of Public Health, Exercise Biology, Aarhus University, Aarhus, Denmark; ^3^Swedish Winter Sports Research Centre, Department of Health Sciences, Mid Sweden University, Östersund, Sweden; ^4^School of Sport Sciences, Faculty of Health Sciences, UiT The Arctic University of Norway, Tromsö, Norway; ^5^Department of Health Sciences, Luleå University of Technology, Luleå, Sweden; ^6^Department of Physiology and Pharmacology, Karolinska Institute, Stockholm, Sweden

**Keywords:** myofiber, force-generating capacity, cross-country skiing, myosin heavy chain isoforms, athletes, exercise, *triceps brachii*, *vastus lateralis*

## Abstract

**Introduction:**

Little is known about potential differences in contractile properties of muscle fibers of the same type in arms and legs. Accordingly, the present study was designed to compare the force-generating capacity and Ca^2+^ sensitivity of fibers from arm and leg muscles of highly trained cross-country skiers.

**Method:**

Single muscle fibers of *m. vastus lateralis* and *m. triceps brachii* of eight highly trained cross-country skiers were analyzed with respect to maximal Ca^2+^-activated force, specific force and Ca^2+^ sensitivity.

**Result:**

The maximal Ca^2+^-activated force was greater for myosin heavy chain (MHC) II than MHC I fibers in both the arm (+62%, *P* < 0.001) and leg muscle (+77%, *P* < 0.001), with no differences between limbs for each MHC isoform. In addition, the specific force of MHC II fibers was higher than that of MHC I fibers in both arms (+41%, *P* = 0.002) and legs (+95%, *P* < 0.001). The specific force of MHC II fibers was the same in both limbs, whereas MHC I fibers from the *m. triceps brachii* were, on average, 39% stronger than fibers of the same type from the *m. vastus lateralis* (*P* = 0.003). pCa_50_ was not different between MHC I and II fibers in neither arms nor legs, but the MHC I fibers of *m. triceps brachii* demonstrated higher Ca^2+^ sensitivity than fibers of the same type from *m. vastus lateralis* (*P* = 0.007).

**Conclusion:**

Comparison of muscles in limbs equally well trained revealed that MHC I fibers in the arm muscle exhibited a higher specific force-generating capacity and greater Ca^2+^ sensitivity than the same type of fiber in the leg, with no such difference in the case of MHC II fibers. These distinct differences in the properties of fibers of the same type in equally well-trained muscles open new perspectives in muscle physiology.

## Introduction

The ability of human skeletal muscles to produce force and power repeatedly during physical activity is determined in part by the inherent properties of the single muscle fibers, properties which are dependent on their expression of different myosin heavy chain (MHC) isoforms (Harridge et al., 1996; Allen et al., 2008). MHC is the motor protein of the myosin filament and the human skeletal muscles express three different isoforms: MHC I, MHC IIa, and MHC IIx. The functional significance of the MHC isoform for its contractile characteristics is well established (Schiaffino and Reggiani, 2011), even for hybrid fibers co-expressing MHC isoforms. Generally, MHC I has been shown to be slow contracting, while MHC IIa and MHC IIx fibers are generally faster and more powerful (Schiaffino and Reggiani, 2011). Also, Ca^2+^ regulatory properties of the contractile apparatus of skeletal muscle fibers have been shown to be fiber type dependent in both rodents and humans, i.e., maximum Ca^2+^ activated force, the force-Ca^2+^ relationship and herein Ca^2+^ sensitivity (pCa_50_: Ca^2+^ needed to elicit 50% of maximum force). Thus, in single fibers from untrained and trained individuals as well as from different muscle groups, it has generally been observed that MHC II fibers demonstrate a higher maximum Ca^2+^ activated force in comparison to MHC I fibers, whereas the Ca^2+^ sensitivity is generally higher in MHC I fibers (Hvid et al., 2013a; Lamboley et al., 2015, 2020; Gejl et al., 2016).

Thus, there appears to be a link between contractile function of isolated fibers and their MHC isoform. Yet, despite these general differences between MHC I and II fibers, large variability in contractile properties exist within fibers expressing the same MHC isoform (Bottinelli et al., 1996), obviously linked to the adaptational plasticity of the contractile properties of fibers expressing a specific MHC isoform (Bottinelli et al., 1996). Specifically, considerable functional adaptability within both MHC I and II fibers has been observed in response to acute exercise (Hvid et al., 2013a; Gejl et al., 2016; Lamboley et al., 2020), training (Lynch et al., 1994; Trappe et al., 2001, 2006; Widrick et al., 2002; Malisoux et al., 2006a,b), immobilisation (Hvid et al., 2011, 2013b), and tapering (Trappe et al., 2000, 2006). For instance, prolonged exercise has been shown to acutely compromise the maximum Ca^2+^ activated force in single fibers from highly trained endurance athletes, whereas the Ca^2+^ sensitivity has been shown to be acutely increased in response to high-intensity exercise, probably as a result of the enhanced production of reactive oxygen species and/or reactive nitrogen species (Hvid et al., 2013a; Gejl et al., 2016). This means that the MHC distribution is not explanatory for muscle function itself, and emphasizes the necessity of considering the functional characteristics of the different MHC isoforms (Harridge et al., 1996).

Most studies of single muscle fiber characteristics have been performed in lower limb muscles (i.e., *m. vastus lateralis*), while only a few studies have investigated other muscles including upper body muscles as the deltoid muscle (Trappe et al., 2000). Consequently, little is known about potential limb-differences in single fiber contractile properties. While such physiological comparisons of arms and legs are often hampered by an unequal training status of these limbs, cross-country skiing is nevertheless physiologically demanding for both the upper and lower extremities. Hence, highly trained cross-country skiers provide a unique model for a comparative analysis of mechanistic limb differences. Recent findings from our laboratory have revealed interesting limb differences in metabolic characteristics of such athletes that may reflect adaptations to the specific demands of their sport (Ørtenblad et al., 2018). Specifically, the *m. triceps brachii*, which had a relatively low content of MHC I fibers (40 ± 8%) demonstrated a mitochondrial volume and capillarization similar to or higher than that of *m. vastus lateralis*, which contained a larger relative fraction of MHC I fibers (58 ± 6%) (Ørtenblad et al., 2018). In fact, the MHC II fibers of *m. triceps brachii* tended to have a higher mitochondrial volume than those of *m. vastus lateralis*, which could be interpreted as a compensatory mechanism to the lower fraction of oxidative MHC I fibers. While clear metabolic differences were evident between specific MHC isoforms of trained muscles in the upper and lower body of those particular athletes, it remains unclear if this is also the case regarding the maximum Ca^2+^ activated force and the force-Ca^2+^ relationship.

The present study was designed to compare the contractile properties, i.e., force-generating capacity and Ca^2+^ sensitivity, (1) between the same fiber type in trained muscles from the upper or lower limbs, as well as (2) between fiber types within limbs. For this purpose, we examined MHC I and MHC II fibers from leg muscle (*m. vastus lateralis*) and arm muscle (*m. triceps brachii*) of highly trained cross-country skiers with a remarkably well-trained lower and upper body. We hypothesized that fibers from the equally trained arm and leg muscles would demonstrate similar contractile properties within the specific fiber types.

## Materials and Methods

### Study Design

Resting muscle biopsies were extracted from arm and leg muscles of eight elite male cross-country skiers who competed in sprint and distance races at the national or international level (age 24 ± 4 years, body mass 79 ± 7 kg, V̇O_2_max diagonal skiing (DS) 66 ± 3 ml⋅kg^–1^⋅min^–1^, V̇O_2_max double poling (DP) 64 ± 3 ml⋅kg^–1^⋅min^–1^). Single muscle fibers isolated from the muscle biopsies were analyzed with respect to their contractile properties and typed based on their MHC isoform expression. The project was pre-approved by the Regional Ethics Review Board in Umeå, Sweden (#2013-59-31), and all subjects were fully informed of any risk associated with the experiments before providing written consent to participate. The study was part of a larger project, with the remaining muscle tissue being used for other purposes (Andersson et al., 2016, 2017; Gejl et al., 2016, 2017). One part of the data has been published previously (*m. triceps brachii* single muscle fiber contractile properties) (Gejl et al., 2016), but is included here for a limb comparison purpose.

### Maximal Oxygen Uptake (VO_2_max)

V̇O_2_max was measured using an ergospirometry system (AMIS 2001 model C, Innovision A/S, Odense, Denmark). The gas analyzers were calibrated with a mixture of 16.0% O_2_ and 4.0% CO_2_ (Air Liquide, Kungsängen, Sweden) and calibration of the flowmeter was performed at low, medium, and high flow rates with a 3 L air syringe (Hans Rudolph, Kansas City, MO, United States). V̇O_2_max was determined twice in connection with an incremental roller ski treadmill test using the diagonal skiing technique in one test and the DP technique in the other test, in a randomized order. The treadmill inclination was 1° (DP) or 7° (DS) throughout the tests and the speed started at 21 km/h (DP) or 9 km/h (DS) and was increased by 1 km/h (DP) or 0.5 km/h (DS) every 60 s (DP) or 45s (DS) until exhaustion. The average V̇O_2_ of the three highest 10s consecutive values was defined as V̇O_2_max.

### Muscle Biopsies

In the current study, the skiers were asked to refrain from moderate-to-high intensity exercise for 48 h prior to the extraction of the muscle biopsies and. we analyzed fibers from resting biopsies obtained in one arm muscle (the distal part of the lateral head of *m. triceps brachii*) and one leg muscle (the mid-section of *m. vastus lateralis*). The Bergström needle biopsy technique was used to obtain muscle samples (Bergstrom, 1975). Immediately following the biopsy extraction, the muscle sample was placed on a filter paper on an ice-cooled ∼0°C petri dish and divided into five specimens of which one part was used for single muscle fiber analyses. This part was quickly placed in storage solution (see below) and initially stored for 24 h at 4°C and subsequently washed in the storage solution and stored at −20°C until the day of analysis. For determination of whole muscle MHC distribution, a segment was weighed and homogenized in 10 volumes (wt/vol) of ice-cold buffer (300mM sucrose, 1 mM EDTA, 10 mM NaN_3_, 40 mM Tris-base, and 40 mM histidine at pH 7.8) in a 1-ml glass homogenizer with a glass pestle (Kontes Glass Industry, Vineland, NJ, United States).

### Muscle Fiber Preparation and Cross-Sectional Area

The single fiber analysis has been described in detail elsewhere (Hvid et al., 2011). Briefly, a small bundle of muscle fibers from each biopsy (∼40 fibers) was blotted and placed in cold paraffin oil (0–5°C) and single muscle fibers were randomly selected from three different sections of the muscle bundle and then isolated under a dissecting microscope (Stemi 2000-C, Zeiss, Germany). A loop of surgical silk (Genzyme, MA, United States) was attached to each end of an isolated fiber, and small metal pins were used to carefully stretch and fix the fiber at a length where its curved appearance disappeared (i.e., slack length). Then a picture of the fiber was taken (Canon, Powershot A80 digital camera and LA-DC583 conversion lens adapter, Japan) in order to calculate the cross sectional area (CSA) based on the mean of three diameter measurements along the fiber length (iTEM software, version 5.0, Olympus, Germany), assuming a cylindrical shaped fiber. No corrections were made for fiber swelling.

### Solutions

The storage solution contained (in mM): 5 EGTA, 2 Na_2_-ATP, 2 MgCl_2_, 150 K-propionate, and 50% vol/vol glycerol. For the single fiber analysis, each chemically skinned fiber was exposed to solutions containing different concentrations of free Ca^2+^ ([Ca^2+^]_*free*_), mimicking the intracellular environment. The solutions were made by mixing two different stock solutions with strongly buffered Ca^2+^ capacity by EGTA and having either high Ca^2+^ (Ca-EGTA solution) or zero Ca^2+^ (EGTA or relaxing solution) as previously described (Stephenson and Williams, 1981). The EGTA solution consisted of (in mM): 90 HEPES, 10.3 MgO, 50 EGTA, 8 Na_2_-ATP, 10 Na_2_-CrP and the Ca^2+^-EGTA solution consisted of: 90 HEPES, 8.1 MgO, 50 EGTA, 48.5 CaCO_3_, 8 Na_2_-ATP, 10 Na_2_-CrP. The EGTA/Ca-EGTA solutions were mixed in appropriate volumes in order to obtain solutions with different [Ca^2+^]_*free*_ (Stephenson and Williams, 1981): pCa > 9.0 (relaxing solution), pCa 6.7, 6.4, 6.2, 5.9, 5.5 (submaximal activating solutions), and pCa 4.7 (maximal Ca^2+^ activating solution), where pCa = −log [Ca^2+^]. The same stock solutions were used for all single fiber analyses. All solutions had an osmolality of 298 ± 8 mosmol/L, pH 7.10 ± 0.01, and a calculated free [Mg^2+^] of 1 mM (Lamb and Stephenson, 1994).

### Force Recordings

Force transducers were calibrated prior to use and all measurements were carried out at room temperature (22.1 ± 0.0°C) using a customized setup. Each single fiber was mounted to a length-adjustable force measurement setup (AE801, Memscap, France), and initially chemically skinned for ∼60 s in a relaxing solution containing 1% of Triton-X. Next, the fiber was washed in the relaxing solution and adjusted to 120% of slack length, to optimize conditions for force generation, and kept at this length for ∼60 s to ensure that any passive tension plateaued. Fibers were then immersed into Ca^2+^ solutions to obtain maximal Ca^2+^ activated force (first measurement), force-Ca^2+^ relationship, and maximal Ca^2+^ activated force (second measurement). Force recordings were sampled at 1000 Hz and stored for later analysis by custom-made software (LabView 8.0, National Instruments, Austin, Texas, USA). Maximal Ca^2+^ activated force is expressed in milli-newtons (mN) and specific force (SF) in kN⋅m^–2^ (i.e., force normalized to CSA). For each fiber, force production at each pCa (i.e., 6.7, 6.4, 6.2, 5.9, 5.5, and 4.7) was expressed relative to the maximal force. A sigmoidal curve was then fitted for each fiber by non-linear regression and based on the Hill equation, the Ca^2+^ sensitivity ([Ca^2+^] needed to elicit 50% of maximal force, pCa_50_) and the Hill coefficient (representing the slope of the relationship) were derived (GraphPad Prism 6.0, GraphPad Software Inc., San Diego, California, USA). Since pCa_50_ is −log[Ca^+2^], a lower pCa50 indicates that more Ca^2+^ is needed for at given relative force output, i.e., lower Ca^2+^ sensitivity. Mean force-Ca^2+^ relationship curves were created by plotting the mean of the relative force from all fibers at each pCa (i.e., 6.7, 6.4, 6.2, 5.9, 5.5, and 4.7) using sigmoidal curve fitting. Also, we specifically examined Ca^2+^ activated force at pCa 5.9, as this concentration is within the physiological range during exercise (Bruton et al., 2003; Olsson et al., 2020).

### Myosin Heavy Chain Isoform Determination

Following measurements of contractile function, each single fiber was placed in an Eppendorf tube containing 20 μL of sample buffer, boiled for 3 min, and stored at –80°C until further examination of the MHC composition by SDS-PAGE analysis (Danieli Betto et al., 1986). Gels were silver stained using a commercial kit (Amersham Biosciences AB, Uppsala, Sweden). MHC I, IIa, IIx, or mixed isoforms (I/IIa, IIa/IIx) were determined by comparing protein band migration to a standard myosin extract run in one or more lanes on the gel (Hvid et al., 2011). The whole muscle MHC composition was determined using gel electrophoresis as described previously (Ørtenblad et al., 2011). In brief, muscle homogenate (80 μL) was mixed with a sample-buffer (10% glycerol, 5% 2-mercaptoethanol and 2.3% SDS, 62.5 mM Tris, and 0.2% bromophenol blue at pH 6.8.), boiled in water for 3 min, and loaded with three different quantities of protein (10–40μL) on a SDS-PAGE gel [6% polyacrylamide (100:1 acrylmid:bis-acrylmid), 30% glycerol, 67.5 mM Tris-base, 0.4% SDS and 0.1 M glycine]. Gels were run at 80 V for at least 42hrs at 4°C and MHC bands were made visible by staining them with Coomassie. The gels were scanned (Linoscan 1400 scanner; Linoscan Heidelberg, Germany) and MHC bands were quantified densiometrically (Phoretix 1D, non-linear; Phoretix International Ltd., Newcastle, United Kingdom) as an average of the two to three loaded protein amounts, giving clear MHC bands. MHC II was identified by western blotting, using a monoclonal antibody (M 4276; Sigma, St. Louis, MO, United States), with the protocol Xcell IITM (Invitrogen, Carlsbad, CA, United States).

### Single Fiber Analyses

In total, 173 single muscle fibers were successfully prepared, analyzed, and separated based on MHC isoforms. As 35 of these were hybrid isoforms (MHC I/II or II/IIx), these hybrid isoforms were excluded from further analysis. Since no MHC IIx fibers were identified in the arm muscle, and only four were found in the leg muscle, this isoform was also excluded from the analysis. All values outside the mean ± 2SD for specific force were regarded as outliers and omitted from the analysis (*n* = 6). Consequently, maximum Ca^2+^ activated force was determined in 132 fibers, while the F-pCa relationship was determined in 119 fibers, due to the rupture of 13 fibers during this analysis (for exact numbers in each group see Table 1 and figure legends). In these cases, the initial measurement of the maximal Ca^2+^ activated force was included.

**TABLE 1:** The pCa_50_, Hill coefficient and cross-sectional area (means ± SD) of MHC I and II muscle fibers isolated from *m. triceps brachii* and *m. vastus lateralis* of eight highly trained cross-country skiers.

	**MHC I fibers**		**MHC II fibers**	
	***m. triceps brachii***	***m. vastus lateralis***	***m. triceps brachii***	***m. vastus lateralis***
*n* (individual fibers)	27	30	48	14
pCa_50_	5.94 ± 0.15*	5.87 ± 0.04	5.91 ± 0.12	5.87 ± 0.05^§^
Hill coefficient	3.20 ± 1.10^#^	7.21 ± 4.90^#^	8.28 ± 8.42*	12.15 ± 4.41^§^
Cross sectional area (mm^2^)	7619 ± 2566^*#^	8970 ± 1906	8807 ± 2142	8211 ± 1382^§^

*P < 0.01 compared to corresponding value for the same MHC isoform in m. vastus lateralis. ^#^P < 0.001 compared to corresponding value of MHC II fibers in the same muscle. ^§^ Significant MHC × limb interaction (pCa50: P = 0.04, Hill: P < 0.0001, CSA: P < 0.0001.

### Statistical Analysis

A linear mixed model was used for statistical analysis (STATA 10.1, StataCorp, College Station, TX, United States) (Hvid et al., 2011). If data were not normally distributed, log, square root, or inverse square root transformations were performed (according to the STATA function *ladder*). Properties of single fiber contractile function were analyzed with *subject ID* and *fiber type* (I, II) as random effects, and with *limb* (Arm, Leg) and *fiber type* (I, II) as fixed effects. Specifically, we compared (1) contractile properties of each fiber type *per se* between limbs in one analysis, and (2) contractile properties between fiber types within each limb separately in another analysis. Data are given as mean ± standard deviation (SD), and the level of statistical significance was *P* < 0.05.

## Results

### Myosin Heavy Chain Distribution in Arm and Leg Muscles

The fraction of MHC I fibers was significantly higher in homogenate from the *m. vastus lateralis* than from the *m. triceps brachii* (51 ± 12% vs. 39 ± 6%, *P* = 0.03) and, accordingly the relative content of MHC II fibers was higher in the *m. triceps brachii* in comparison to *m. vastus lateralis* (61 ± 6% vs. 49 ± 12%, *P* = 0.03). The MHC distribution from the arm muscle has been reported previously (Gejl et al., 2020).

### Single Fiber CSA, Maximal Force-Generating Capacity, and Specific Force

Significant MHC × limb interactions were seen for both CSA (*P* = 0.04), the single fiber force generating capacity (*P* < 0.0001), and the single fiber specific force generating capacity (*P* < 0.0001).

#### Myosin Heavy Chain Differences

**FIGURE 1:**
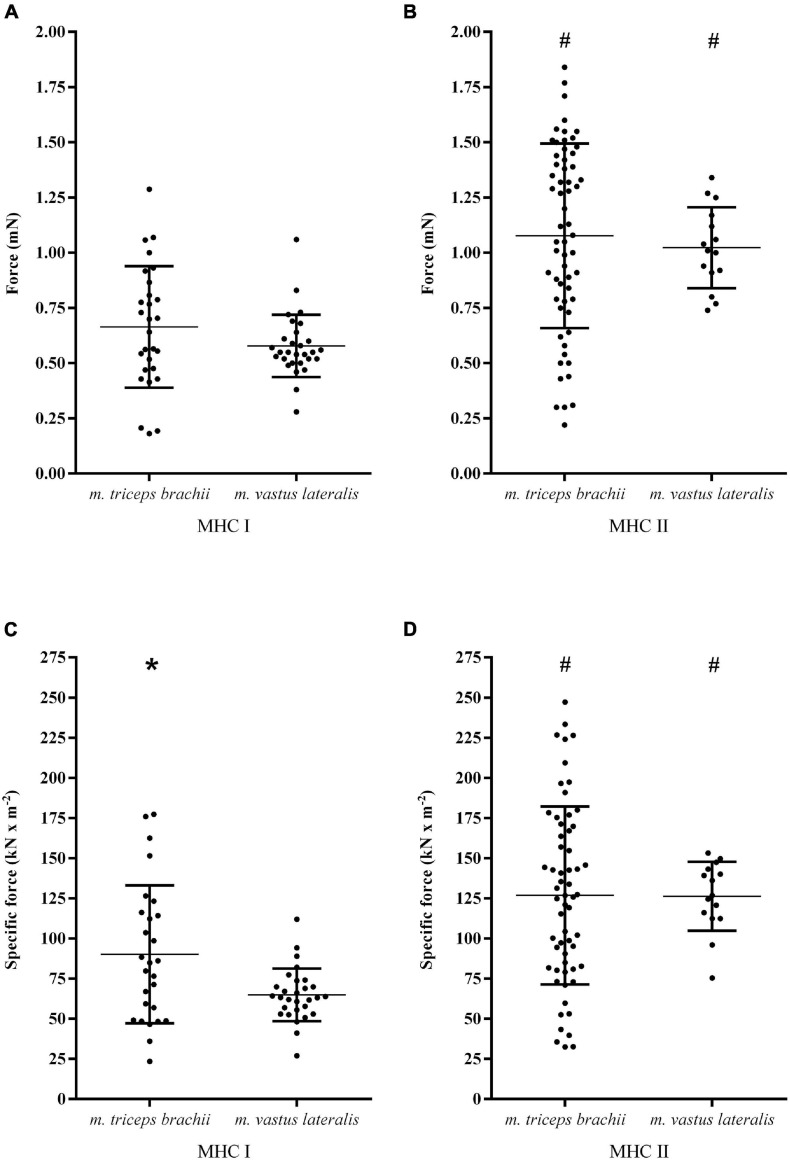
The maximal Ca^2+^-activated force and specific force (i.e., force per CSA) in MHC I **(A,C)** and MHC II fibers **(B,D)** isolated from the *m. triceps brachii* and *m. vastus lateralis* of eight highly trained cross-country skiers. The dots represent values for individual fibers, the horizontal lines the mean value and vertical lines the standard deviations. MHC I fibers: *n* = 28 in *m. triceps brachii*; *n* = 29 in *m. vastus lateralis*. MHC II fibers: *n* = 60 in *m. triceps brachii*; *n* = 15 in *m. vastus lateralis*. There were significant MHC × limb interactions for both maximal Ca^2+^ activated force (*P* < 0.0001) and specific force (*P* < 0.0001). ^#^Significantly different from MHC I fibers from the same muscle (*P* = 0.002); *Significantly different from the same MHC isoform of *m. vastus lateralis* (*P* = 0.003).

As shown in Figure 1 and Table 1, clear differences in both size and properties between MHC I and MHC II fibers within the arm and leg muscles were observed. The CSA of MHC II fibers was 16% greater in comparison to MHC I fibers in the *m. triceps brachii* (*P* = 0.02), while MHC I and II fibers demonstrated a comparable CSA in *m. vastus lateralis* (Table 1). Compared to MHC I fibers, the maximal Ca^2+^-activated force of MHC II fibers was on average 62% (1.08 ± 0.42 mN vs. 0.66 ± 0.28 mN, *P* < 0.001) and 77% (1.02 ± 0.18 mN vs. 0.58 ± 0.14 mN, *P* < 0.001) higher in the *m. triceps brachii* and *m. vastus lateralis*, respectively (Figures 1A,B). Also, the specific force of MHC II fibers was 41% (127 ± 55 kN⋅m^–2^ vs. 90 ± 43 kN⋅m^–2^, *P* = 0.002) and 95% (126 ± 22 kN⋅m^–2^ vs. 65 ± 16 kN⋅m^–2^, *P* < 0.001) greater than in the MHC I fibers of the arm and leg muscles, respectively (Figures 1C,D).

#### Limb Differences

The CSA of MHC I fibers was on average 15% smaller in the *m. triceps brachii* in comparison to those from the *m. vastus lateralis*, while there was no CSA difference between MHC II fibers from the two muscles (Table 1). With respect to force generation, the MHC I fibers in the *m. triceps brachii* demonstrated a numerically higher maximal Ca^2+^ activated force in comparison to the fibers from *m. vastus lateralis* (+15%, *P* = 0.12). Moreover, the specific force of MHC I fibers from the *m. triceps brachii* proved to be significantly higher than observed in MHC I fibers from the leg muscle (+39%, *P* = 0.003) (Figure 1). By contrast, there were no limb differences in the force-generating capacities of MHC II fibers (Figure 1).

### Single Fiber Ca^2+^ Sensitivity and the Hill Coefficient

Significant MHC × limb interactions were observed for the Ca^2+^-sensitivity (*P* = 0.04), the force generation at pCa 5.9 (*P* = 0.003), and the Hill coefficient (*P* < 0.0001).

#### Myosin Heavy Chain Differences

**FIGURE 2:**
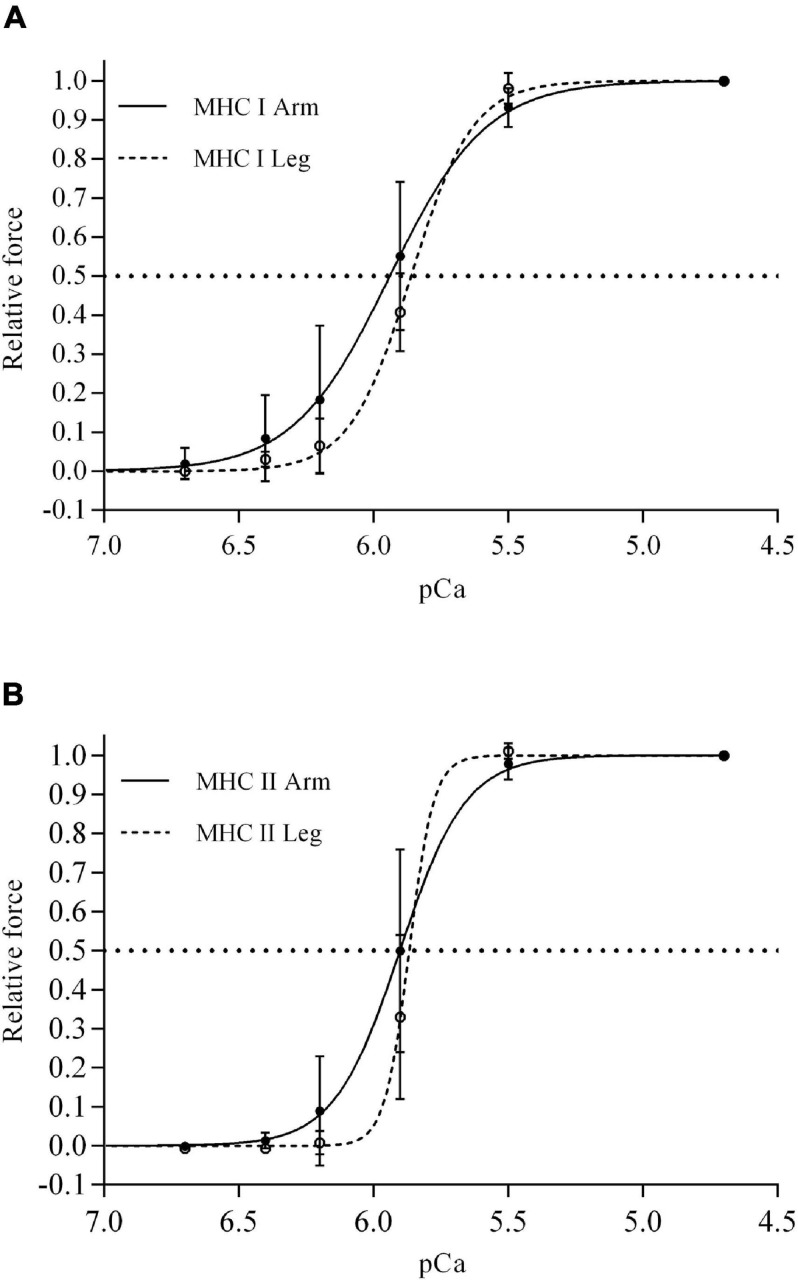
The mean force–Ca^2+^ relationship for single MHC I **(A)** and MHC II fibers **(B)** isolated from *m. triceps brachii* (continuous lines) and *m. vastus lateralis* (discontinuous lines) of eight highly trained cross-country skiers. The horizontal lines indicate pCa_50_ (i.e., 50% of maximal force), the dots are the mean relative force generation at different [Ca^2+^] and the associated vertical lines standard deviations. There were significant MHC × limb interactions for both Ca^2+^ sensitivity (*P* = 0.04), force generation at pCa 5.9 (*P* = 0.003), and the Hill coefficient (*P* < 0.0001). Exact values of pCa_50_ and the slope of the curves (i.e., Hill coefficient) are documented in Table 1.

The Ca^2+^ sensitivity (pCa_50_) was not different between fiber types in neither *m. triceps brachii* nor *m. vastus lateralis* (Table 1 and Figure 2). In contrast, the slope of the force-pCa^2+^ relationship was significantly steeper (i.e., higher Hill coefficient) in the MHC II fibers compared to the MHC I fibers of both *m. triceps brachii* (+159%, *P* < 0.0001) and *m. vastus lateralis* (+69%, *P* < 0.0001) (Table 1).

#### Limb Differences

The Ca^2+^ sensitivity was on average higher in MHC I fibers from *m. triceps brachii* in comparison to the sensitivity observed in MHC I fibers from the *m. vastus lateralis* (*P* = 0.007) (Figure 2 and Table 1), i.e., 20% less Ca^2+^ needed to obtain half maximum Ca^2+^ activated force (1.34 vs. 1.12 μM Ca^2+^). In contrast, pCa_50_ of the MHCII fibers was not different between limbs. At pCa 5.9 (i.e., [Ca^2+^] = 1.3 μM), which is likely within the physiological range during contractions in intact fibers (Bruton et al., 2003; Olsson et al., 2020), and close to pCa50 in skinned fibers (Hvid et al., 2013a; Gejl et al., 2016; Lamboley et al., 2020), the relative force generation of MHC I fibers was significantly different between *m. triceps brachii* and *m. vastus lateralis;* 55 and 41% of the maximal force production, respectively (*P* < 0.001) (Figure 2). A similar limb difference was observed in MHC II fibers, where the force generation at pCa 5.9 elicited 50 and 34% of maximal force production in fibers from *m. triceps brachii and m. vastus lateralis*, respectively (*P* = 0.02) (Figure 2).

At pCa 5.9 the absolute force production in the MHC I fibers was also greater in the arms than in the legs (0.38 ± 0.27 mN vs. 0.23 ± 0.08 mN, respectively, *P* < 0.01), whereas the specific force was not different (53 ± 42 kN⋅m^–2^ vs. 49 ± 19 kN⋅m^–2^, respectively, *P* = 0.61). Similarly, the absolute force production of the MHC II fibers at pCa 5.9 was higher in the arms in comparison to the legs (0.51 ± 0.37 mN vs. 0.29 ± 0.19 mN, respectively, *P* = 0.04), whereas there was no difference between limbs with respect to the specific force (63 ± 57 kN⋅m^–2^ vs. 44 ± 32 kN⋅m^–2^, respectively, *P* = 0.24).

## Discussion

Elite cross-country skiing includes prolonged training sessions that involve a high amount of low-to-moderate intensity whole-body exercise, which places heavy demands on skeletal muscle contractile function of both upper and lower extremities. Accordingly, highly trained cross-country skiers comprise a unique model for characteristically comparisons of trained muscles from different limbs. For the first time, the present study compares contractile properties of single muscle fibers obtained from two trained muscles that are both highly active during cross-country skiing, i.e., *m. vastus lateralis* and *m. triceps brachii* (Holmberg et al., 2005). Here, we demonstrate clear limb differences in contractile properties of single muscle fibers of the same MHC-isoform. Thus, the present findings reveal that the MHC I fibers from *m. triceps brachii* demonstrate a higher Ca^2+^ sensitivity and a higher specific force-generating capacity in comparison to MHC I fibers of *m. vastus lateralis*. Although limb differences were less consistent in MHC II fibers, these fibers showed a higher force production at a submaximal and physiologically relevant Ca^2+^ concentration (pCa 5.9) in the arm muscle compared to leg muscle.

The perception of MHC I fibers as slow, but resistant to fatigue and MHC II fibers as glycolytic, fast, and vulnerable to fatigue has been the reigning dogma. While this dichotomous fiber type separation may be valid for some parameters such as the muscle fiber shortening velocity and production of power (Trappe et al., 2001, 2006; Harber et al., 2004; Luden et al., 2012), it is less clear with regard to the force-generating capacity of trained muscle fibers. We have previously shown that, on average, MHC II fibers exhibit a greater maximal force production than MHC I fibers in the *m. vastus lateralis* of elite endurance athletes (Hvid et al., 2013a), which was also the case in both the arm and leg muscle in the present study. Since MHC differences in the force-generating capacity observed here remained even after normalization for CSA, these were likely explained by qualitative differences, such as higher force production per cross-bridge, and/or a higher number of cross-bridge attachments and/or more attachments in the strong binding state in the MHC II fibers (Fitts et al., 1991; Li and Larsson, 2010; Hvid et al., 2017). At the same time, other investigators have only shown a numerically yet non-significantly higher maximal Ca^2+^ activated force produced by MHC II fibers in trained individuals (Trappe et al., 2001, 2006; Harber et al., 2004; Luden et al., 2012). In less trained individuals, this inconsistency also exists with fiber type differences observed in some but not all studies – *for review see* (Jee and Kim, 2017). Reasons for these slight inconsistencies between studies in trained athletes are not clear but may be related to methodological differences (e.g., skinning process, temperature or the composition of the activating solutions), a lack of statistical power needed to detect a difference, different sport-specific demands, or different muscles of investigation.

Interestingly, the observed MHC differences in the force-generating capacity were dependent on the muscle being investigated in the present study. While we observed a clear distinction between MHC I and II fibers from *m. vastus lateralis*, this was not the case in fibers from *m. triceps brachii*. In this muscle, there appeared to be a clear overlap between MHC I and II isoforms with respect to maximum Ca^2+^ activated force and the specific force (Figure 1). This was accompanied by a much larger variation in both the force-generating capacities and the muscle fiber cross-sectional areas in both MHC isoforms in *m. triceps brachii* (Table 1). Despite this heterogeneity within both MHC isoforms, MHC I fibers from *m. triceps brachii* were on average stronger than MHC I fibers from *m. vastus lateralis*, which supports the existence of functional variability between muscle fibers expressing the same MHC isoform (Harridge et al., 1996; Ørtenblad et al., 2018), yet emphasizes that muscle group differences likely exist. Although speculative, the differences observed here in the functional homogeneity of fibers between muscles could, at least in part, reflect the different demands placed on these two muscles in connection with cross-country skiing. Supporting this idea, several studies have reported considerable plasticity in single fiber contractile properties in response to use and disuse, i.e., following immobilization (Hvid et al., 2011, 2013b), acute exercise (Hvid et al., 2013a; Gejl et al., 2016; Lamboley et al., 2020), training (Malisoux et al., 2006a; Trappe et al., 2006), and tapering (Trappe et al., 2001). During classic cross-country skiing, DP is the only sub-technique where propulsion solely is generated during the poling phase (i.e., via the upper-body), and this sub-technique has become gradually more important over the last decade(s) to overall performance in cross-country skiing. Moreover, both the level of muscle activation and contraction characteristics is likely to differ between arms and legs in cross-country skiing (Björklund et al., 2010). For instance, in DP, both upper- and lower-body muscles are exposed to endurance-like demands by repetitive contractions for several minutes or hours where the range of motion, angular velocity and force production at the elbow joint are all greater than at the hip and knee joints (Holmberg et al., 2005).

We observed that the MHC I fibers from *m. triceps brachii* were on average slightly stronger in comparison to those of *m. vastus lateralis*, and we also observed that a relatively large group of MHC II fibers from *m. triceps brachii* was extraordinarily strong in comparison to those of the leg muscle. Although single fiber power production was not measured in the present study, these strong fibers may support the need for high angular velocities at the elbow joint, reflecting adaptation to years of highly specialized training for cross-country skiing. The higher force generating capacity in the MHC I fibers from the arm muscle could theoretically be explained by either a higher number of cross-bridges per fiber area or intrinsic adaptations leading to a higher force per cross-bridge or a higher number of cross-bridge attachments in the strong binding state (Li and Larsson, 2010). Previous studies have shown that the single fiber myosin content adapts in response to immobilization and re-activation (Hvid et al., 2017), and accordingly, the higher force generating capacity in the fibers from the arm could possibly be explained by a higher myosin protein content in these fibers. However, changes in the single fiber myosin content in response to training in athletes remains to be investigated.

In another group of highly trained cross-country skiers, a study from our laboratory recently investigated differences in the metabolic profiles between muscle fibers from arm and leg muscles (Ørtenblad et al., 2018). Here it was demonstrated that the total volume of mitochondria per volume of myofiber was similar between *m. triceps brachii* and *m. vastus lateralis* despite significantly more type II fibers in the arm muscle. Also, the muscle fiber capillary density was similar between type I and II fibers within each limb, but significantly higher in the type II fibers of the arm muscle in comparison to the type II fibers of the leg muscle. Together with the present data, it seems that neither metabolic nor contractile characteristics of a given fiber type (MHC isoform) are fixed, but to some extent plastic in response to the stimuli to which it is routinely exposed (e.g., training, immobilization etc.).

The Ca^2+^ sensitivity of skeletal muscle fibers, was on average higher in the MHC I fibers from *m. triceps brachii* in comparison to the MHC I fibers from *m. vastus lateralis* (i.e., 20% less Ca^2+^ needed to elicit 50% of maximum force in fibers from the arm muscle) (Table 1 and Figure 2). Also at pCa 5.9, which is located on the steep portion of the sigmoidal force–pCa curve and within the physiological range that exists during exercise (Bruton et al., 2003; Olsson et al., 2020), higher absolute force was elicited in both the MHC I and II fibers from the arm muscle in comparison to the leg muscle. The functional impact of a higher Ca^2+^ sensitivity may be particularly relevant in situations where the release of Ca^2+^ from the sarcoplasmic reticulum (SR) is compromised as observed during exhausting exercise (Gejl et al., 2014, 2020). Thus, a reduction in the SR Ca^2+^ release during exercise reduces the available amount of intracellular Ca^2+^ to interact with troponin C, which inhibits cross-bridge cycling and potentially leads to muscle fatigue (Place et al., 2010). Accordingly, fibers with a high Ca^2+^ sensitivity will be able to maintain a relatively high force production when the Ca^2+^ release and consequently the intracellular [Ca^2+^] is reduced as during exhaustive exercise. The higher Ca^2+^ sensitivity may also mean a lower need of neural stimulation, and hence reduce the sense of effort to develop a certain submaximal force.

Calcium sensitivity can be modulated by several factors, including temperature, pH, phosphorylation of myosin light chains, as well as oxidation and/or nitrosylation of proteins in the contractile apparatus (MacIntosh, 2003; Mollica et al., 2012; Gejl et al., 2016). However, these modulations normally occur as an acute response to repetitive muscle contractions and fatigue, and since the biopsies were obtained during rest in the present study, the observed limb differences in the Ca^2+^ sensitivity must be explained by long-term persistent adaptations to exercise. While underlying mechanisms were not investigated in the present study, we speculate that selective adaptations in the expression or functioning of regulatory proteins and isoforms hereof [i.e., troponin I (TnI), troponin C (TnC), and tropomyosin (Tm)] were in part associated with our findings, as these are central proteins involved in the force-Ca^2+^ relationship (MacIntosh, 2003).

The Hill coefficient, which is proportional to the maximum steepness of the F–pCa curve, is generally higher (steeper F-pCa relationship) in MHC II fibers compared to MHC I fibers (Lynch et al., 1994; Hvid et al., 2013a; Gejl et al., 2016; Lamboley et al., 2020). This is confirmed here in both *m. triceps brachii* and *m. vastus lateralis* (Figure 2 and Table 1). Interestingly, there was a general higher Hill coefficient in leg muscle compared to arm for both fiber types. The Hill coefficient refers to the number of Ca^2+^ ions and cooperativity between the Ca^2+^-regulatory sites in the proteins involved in the process of force generation. This is both dependent on the contractile apparatus protein isoform and may also be separately affected by the many and various means by which oxidation can modify contractile force oxidants (Wilson et al., 1991; Lamb and Posterino, 2003). However, the mechanism(s) of the limb differences in Hill coefficient within the given MHC isoform remains to be established.

In conclusion, the present study provides novel findings of inter-limb differences in human single muscle fiber contractile properties. In general, contractile properties of fibers from *m. triceps brachii* exhibited a force-generating capacity and Ca^2+^ sensitivity greater than observed in the fibers from *m. vastus lateralis*. Differences between limbs were particularly evident in MHC I fibers. This divergence in contractile properties between muscle fibers expressing the same MHC isoform may reflect adaptations to specific demands placed on the two muscles during cross-country skiing, and the observed differences may have important implications for muscle function and performance.

## Data Availability Statement

The raw data supporting the conclusions of this article will be made available by the authors, without undue reservation, upon request.

## Ethics Statement

The studies involving human participants were reviewed and approved by the Regional Ethics Review Board in Umeå, Sweden (#2013-59-31). The patients/participants provided their written informed consent to participate in this study.

## Author Contributions

KG, LH, EA, NØ, and H-CH were responsible for the conception and design of the study. KG, LH, NØ, and RJ were responsible for the acquisition, analysis, and interpretation of the data. KG, LH, RJ, EA, H-CH, and NØ were responsible for drafting the manuscript and revising it critically for important intellectual content. All authors have approved the final version of the manuscript. All persons designated as authors qualify for authorship.

## Conflict of Interest

The authors declare that the research was conducted in the absence of any commercial or financial relationships that could be construed as a potential conflict of interest.
